# Impaired Hippocampal Neurovascular Coupling in a Mouse Model of Alzheimer’s Disease

**DOI:** 10.3389/fphys.2021.715446

**Published:** 2021-08-12

**Authors:** Lin Li, Xin-Kang Tong, Mohammadamin Hosseini Kahnouei, Diane Vallerand, Edith Hamel, Hélène Girouard

**Affiliations:** ^1^Department of Pharmacology and Physiology, Faculty of Medicine, Université de Montréal, Montréal, QC, Canada; ^2^Groupe de Recherche sur le Système Nerveux Central (GRSNC), Université de Montréal, Montréal, QC, Canada; ^3^Laboratory of Cerebrovascular Research, Montreal Neurological Institute, McGill University, Montréal, QC, Canada; ^4^Centre Interdisciplinaire de Recherche sur le Cerveau et l’Apprentissage (CIRCA), Université de Montréal, Montréal, QC, Canada; ^5^Centre de Recherche de l’Institut Universitaire de Gériatrie de Montréal, Montréal, QC, Canada

**Keywords:** neurovascular coupling, Alzheimer’s disease, hippocampus, astrocyte, two-photon imaging

## Abstract

Alzheimer’s disease (AD), the most common form of dementia, is characterized by neuronal degeneration and cerebrovascular dysfunction. Increasing evidence indicates that cerebrovascular dysfunction may be a key or an aggravating pathogenic factor in AD. This emphasizes the importance to investigate the tight coupling between neuronal activity and cerebral blood flow (CBF) termed neurovascular coupling (NVC). NVC depends on all cell types of the neurovascular unit within which astrocytes are important players in the progression of AD. Hence, the objective of this study was to characterize the hippocampal NVC in a mouse model of AD. Hippocampal NVC was studied in 6-month-old amyloid-beta precursor protein (APP) transgenic mice and their corresponding wild-type littermates using *in vivo* laser Doppler flowmetry to measure CBF in area CA1 of the hippocampus in response to Schaffer collaterals stimulation. *Ex vivo* two-photon microscopy experiments were performed to determine astrocytic Ca^2+^ and vascular responses to electrical field stimulation (EFS) or caged Ca^2+^ photolysis in hippocampal slices. Neuronal synaptic transmission, astrocytic endfeet Ca^2+^ in correlation with reactive oxygen species (ROS), and vascular reactivity in the presence or absence of Tempol, a mimetic of superoxide dismutase, were further investigated using electrophysiological, caged Ca^2+^ photolysis or pharmacological approaches. Whisker stimulation evoked-CBF increases and *ex vivo* vascular responses to EFS were impaired in APP mice compared with their age-matched controls. APP mice were also characterized by decreased basal synaptic transmission, a shorter astrocytic Ca^2+^ increase, and altered vascular response to elevated perivascular K^+^. However, long-term potentiation, astrocytic Ca^2+^ amplitude in response to EFS, together with vascular responses to nitric oxide remained unchanged. Importantly, we found a significantly increased Ca^2+^ uncaging-induced ROS production in APP mice. Tempol prevented the vascular response impairment while normalizing astrocytic Ca^2+^ in APP mice. These findings suggest that NVC is altered at many levels in APP mice, at least in part through oxidative stress. This points out that therapies against AD should include an antioxidative component to protect the neurovascular unit.

## Introduction

Alzheimer’s disease (AD), the most common form of dementia in the elderly, is characterized by progressive memory decline and deficits. The disease is defined by pathological features such as amyloid β accumulation, hyperphosphorylated tau protein in the form of neurofibrillary tangles, neuronal and synaptic loss, and cerebrovascular dysfunction (Jack et al., 2018). Increasing evidences indicate that cerebrovascular dysfunctions may play a key role in the pathogenesis of AD (Zlokovic, 2011). Since the cerebrovascular deficits develop very early in the AD process, it is detectable in individuals with mild cognitive impairment (MCI) and promote conversion from MCI to AD (Yetkin et al., 2006; Iadecola, 2010; Li et al., 2011). It has been widely reported that neurovascular coupling (NVC), a vasodilatory response to neuronal activity, is impaired in AD patients or animal models (Hock et al., 1997; Iadecola, 2004; Tong et al., 2012; Lourenço et al., 2017). However, the underlying mechanisms of NVC dysruption in the context of AD are still largely unknown.

NVC depends on all the components of the neurovascular unit, which comprises neurons, astrocytes, and vascular cells. Astrocytes are recognized to transduce the neuronal signal into a dynamic intracellular calcium (Ca^2+^) signal, which travels to astrocytic processes (“endfeet”) to modulate local blood flow (Zonta et al., 2003a; Filosa et al., 2004; Mulligan and MacVicar, 2004; Takano et al., 2006). In murine models of AD, the astrocytic Ca^2+^ signaling is disturbed, with elevated resting Ca^2+^ levels, increased frequency of spontaneous Ca^2+^ spikes or abnormal Ca^2+^ waves (Takano et al., 2007; Kuchibhotla et al., 2009). However, the astrocytic Ca^2+^ signaling in relation to the vascular responses in hippocampal NVC has never been investigated in these models.

Reactive oxygen species (ROS) are elevated in astrocytes of APP mice and Aβ-induced ROS is known to alter vascular regulation (Abramov et al., 2004; Tong et al., 2009). Furthermore, antioxidant drugs completely rescue cerebrovascular functions in old AD transgenic mice (Nicolakakis et al., 2008). Thus, we hypothesized that ROS, may impair NVC by altering the communication between neurons, astrocytes and vascular cells in AD mice.

In this study we found that hippocampal NVC is altered at many levels in adult APP mice compared with age-matched controls. Indeed, basal synaptic transmission in hippocampus is attenuated, the astrocytic Ca^2+^ response to electrical field stimulation (EFS) is shorter, the CBF response to Schaffer collaterals stimulation is decreased, and the vascular responses to EFS, to Ca^2+^ uncaging or to elevated perivascular K^+^ are reversed or attenuated. Importantly, Ca^2+^ uncaging-induced ROS production is elevated in APP mice and ROS scavenging rescue NVC impairment while normalizing astrocytic Ca^2+^ signaling in these mice. The data unveil impaired hippocampal NVC in APP mice and, further, they reinforce the great potential for antioxidant therapies to protect the neurovascular unit in AD.

## Materials and Methods

### Animals

All experimental protocols used in this study are in accord with the Canadian Council on Animal Care, the ARRIVE (Animal Research: Reporting of *In Vivo* Experiments) guidelines and approved by the Committee on Ethics of Animal Experiments of the Université de Montréal or the Montreal Neurological Institute at McGill University. Heterozygous transgenic C57BL/6 male and female mice of 6 month old carrying the familial Swedish (K670N, M671L; APPSwe) and Indiana (V717F; APPInd) human APP mutations directed by the (platelet-derived growth factor) PDGF-chain promoter (APP mice, J20 line) (Mucke et al., 2000) and their wild-type (WT) littermates were used. Animals were housed 4/cages in a temperature-controlled room with *ad libitum* access to water and a standard protein rodent diet (4% fat, 14% protein rodent maintenance diet, Harlan Teklad global). Mice were randomly separated into different groups for each experiment.

### Hippocampal CBF Measurements

**FIGURE 1:**
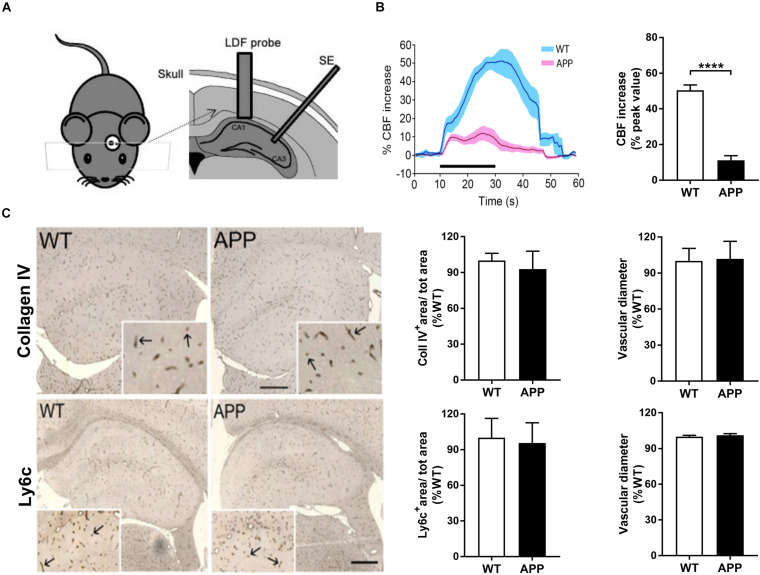
Impaired neurovascular coupling in the hippocampus of APP mice. **(A)** Schematic drawing of the implantation of the laser Doppler flowmetry (LDF) probe and stimulating electrode (SE). **(B)** The increase in hippocampal CBF evoked by Schaffer collaterals stimulation (Scs) is reduced in APP mice compared to their wild-type (WT) controls. Left: Traces of averaged evoked CBF responses acquired before, during, and after Scs (WT, blue and APP purple). SEM is represented by the lighter tone shade surrounding each curve. Right: Bar graph of the peak values of evoked CBF responses in WT (*n* = 6) and APP (*n* = 6) mice, expressed as the percent increase from pre-stimulation (baseline) levels. **(C)** Hippocampal blood vessel surface area and diameter, respectively, immunostained for collagen IV (upper panel) and Ly6c (endothelial marker, lower panel) in 5 μm-thick paraffin sections in APP mice mice did not differ from those measured in WT mice. Values are given in percentage of WT. Inserts show higher magnification of the vessels (arrows). Bar = 400 μm (****p < 0.0001; two-tailed unpaired *t*-test, *n* = 4).

Mice (WT, *n* = 6 and APP, *n* = 6) were anesthetized with ketamine (85 mg/kg, i.m.; Bioniche Life Sciences, CAN) and xylazine (3 mg/kg, i.m.; Haver), and placed in a stereotaxic frame on a heating blanket for maintaining body temperature stable at 37°C. CBF increases were measured in the CA1 area of the hippocampus with laser Doppler flowmetry (Transonic Systems, Ithaca, NY, United States) during stimulation of the Schaffer collaterals in the CA3 area (200 Hz, 50 μA, 0.5 ms, 1 s on/1 s off for 20 s isolated pulse stimulator; A-M Systems, Sequim, WA, United States) (Figure 1A). A 1.0 mm laser Doppler probe was positioned over the CA1 area following insertion through the cerebral cortex via a small opening. Stereotaxic coordinates (Franklin and Paxinos, 1997) were as follows: stimulating electrode (angle: 41 degrees, anterior-posterior (AP): Bregma, −2.3 mm; lateral (L): 3.6 mm and dorso-ventral (V): 1.8 mm; Laser Doppler probe, AP: Bregma, −2.3 mm, L: 1.7 mm and V: 1.0 mm). A total of 4–6 stimulations were performed for each mouse and averaged. CBF was recorded and averaged before, during, and after the stimulation. For illustration, curves were generated representing 1 s average of the evoked CBF, expressed as a percentage change from 30 s pre-stimulus baseline. Stimulation-evoked CBF responses were expressed as the percentage change relative to baseline.

### Immunohistochemical Stainings

Mice (*n* = 4/group) were perfused through the heart with a solution of 4% paraformaldehyde (PFA) in 0.1M phosphate buffer, and their brains were removed and post-fixed in the same solution overnight (4°C). Then, brains were cut in the midline, one hemisphere was processed for paraffin sectioning (5 μm-thick coronal sections) and the other was processed for sectioning on a freezing microtome (25 μm-thick sections) collected in PBS, and kept in anti-freezing solution until use. Paraffin-embedded sections were incubated overnight with a rat anti-Ly6c (1:500, abcam, Canada, an endothelial cell marker), or goat anti-collagen IV (1:300, Millipore-Sigma, Oakville, Canada, a marker of vascular basement membrane). The immunoreactive material was visualized with 3, 3′-diaminobenzidine enhanced with nickel (DAB-Ni). The images were captured on a Panoramic slide scanner (MIRAX SCAN, Zeiss, Oberkochen, Germany), and the total surface area and diameter occupied by blood vessel in the whole hippocampus were measured in each section using MetaMorph6.1r3 software (Universal Imaging, Downingtown, PA). The values were transformed as a ratio of the total hippocampal surface area, and then expressed as a percent of WT controls. Freezing microtome sections were used for triple immunostaining of amyloid β (Aβ) plaques, astrocytes, and blood vessels. The sections were concurrently incubated overnight in rat anti-Ly6c, rabbit anti-GFAP (1:1,000, Dako, Denmark, an astroglial marker) and mouse anti-6E10 (1:1,000, Covance, a marker of Aβ), and the respective reactions were visualized with species-specific donkey anti-rabbit cyanine 2 (Cy2)-, anti-rat Cy3-, and anti-mouse Cy5-conjugated affiniPure secondary antibodies (Jackson ImmunoResearch, West Grove, PA). The associations of astroglial endfeet with local blood vessels or with Aβ plaques were visualized by confocal microscopy (Zeiss LSM 510; Zeiss, Jena, Germany) using simultaneous triple-channel detection with emission intensity of 488 nm (AlexaFluor/Cy2), 543 nm (Cy3), and 640 nm (Cy5).

### Hippocampal Slice Preparation

Mice were sacrificed and brains were quickly removed and placed into ice-cold cutting artificial CSF (aCSF) (250 mM sucrose, 3 mM KCl, 26 mM NaHCO_3_, 1.25 mM NaH_2_PO_4_, 0.5 mM CaCl_2_, 6 mM MgCl_2_, 10 mM glucose, and 400 μM L-ascorbic acid, equilibrated with a 95%O_2_/5%CO_2_ gas at a pH of 7.4). Coronal sections of the hippocampus (180 μm for two-photon imaging or 400 μm for electrophysiological recording) were kept for 20 min at 35°C in recording artificial CSF (125 mM NaCl, 3 mM KCl, 26 mM NaHCO_3_, 1.25 mM NaH_2_PO_4_, 2 mM CaCl_2_, 1 mM MgCl_2_, 10 mM glucose) bubbled with 95%O_2_/5%CO_2_.

### Two-Photon Imaging of Astrocytic Endfoot Ca^2+^ and Arterioles

Hippocampal slices were incubated at 29°C under constant agitation for 1 h in oxygenated aCSF, with the Ca^2+^ indicator Fluo-4 AM (10 μM; Invitrogen) or the ROS indicator CM-H_2_DCFDA (30 μM, 45 min, 25°C), both in the presence of Cremophor EL [0.005% (v/v); Sigma, Oakville, Canada], and pluronic acid F-127 [0.025% (w/v); EMD Calbiochem, Gibbstown, NJ, United States]. For Ca^2+^ uncaging experiments, slices were coloaded with the caged Ca^2+^ compound, 1-[4,5-dimethoxy-2-nitrophenyl]-EDTA-AM (DMNP-EDTA-AM, 10 μM; Interchim) using the same loading conditions.

Imaging was performed with a multiphoton laser scanning upright microscope (BX61WI; Olympus, Tokyo, Japan) coupled to a Ti: Sapphire laser (MaiTai HP DeepSee; Spectra Physics, Santa Clara, CA, United States) and equipped with a 20 × water immersion objective (0.5 numerical aperture; 5 digital zoom factor). Time-lapse images were acquired using the FV10-ASW software (version 3.0; Olympus, Tokyo, Japan) and displayed the arteriole diameter/morphology as visualized by infrared differential interference contrast (IR-DIC) imaging, simultaneously with the free intracellular Ca^2+^ (Fluo-4 AM) in astrocyte endfeet. Fluo-4 AM was excited at 805 nm by the Ti: sapphire laser (100-fs pulses, 0.5 W) laser and fluorescence emission was collected using a 575/150-nm bandpass filter.

For every experiment, morphological criteria were used to distinguish arterioles from venules and capillaries as described earlier (Girouard et al., 2010). Arterioles located 40–80 μm below the cut surface were selected. At the beginning of each experiment, slices were incubated with the thromboxane A2 receptor agonist, U46619 (150 nM; 20 min) to pre-constrict arterioles to a physiological level of arteriolar tone. The constriction level was the same in APP and WT mice (Supplementary Figure 1). An astrocytic endfoot adjacent to the arteriole was then selected at a focal plane corresponding to the highest Fluo-4 AM fluorescence. Images were processed with the Image J software (v.1.45r for Mac OS, NIH, MD Bethesda, United States) and the arteriole luminal diameter was measured adjacently to the selected endfoot on each image. The distance between two points was calculated from a line perpendicular to the arterial walls. The baseline diameter was obtained from the average of 15–20 successive images preceding stimulations. In some experiments, 10 mM K^+^ or the NO donor, sodium nitroprusside (SNP, 100 μM) were added to the aCSF to determine the vascular reactivity to these vasomediators.

### Electrical Field Stimulation of Neuronal Activity and Photolysis of Caged Ca^2+^

Electrical field stimulation (EFS) was used to stimulate neuronal fibers by applying a 50-Hz alternating square pulse of 0.3 ms duration for 2.5 s (50 V) via a concentric bipolar electrode inserted into hippocampal slice near the vessel wall (around 100–150 μm). Multiphoton fluorescence imaging equipped with IR-DIC) microscopy was applied to simultaneously monitor astrocytic endfeet Ca^2+^ (labeled by Fluo4 AM) and arteriol diameter changes before and after EFS. IR-DIC allowed evaluation of the slice integrity through visualization of dead neurons, which was an exclusion criterion. The elevation of astrocytic [Ca^2+^]_i_ by EFS was inhibited by tetrodotoxin (Filosa et al., 2004) (data not shown), a blocker of voltage-dependent sodium channels, confirming neuronal involvement. For Ca^2+^ uncaging experiments, the laser was set at 730 nm, which allows for simultaneous excitation of Fluo-4 and photolysis of the caged Ca^2+^, DMNP-EDTA (Girouard et al., 2010). A 1.5 × 1.5 μm region of interest (ROI) within an endfoot was scanned at a laser intensity 5 × higher than that used for imaging. Based on our preliminary experiments comparing different laser powers over 30 min, the laser power used for Ca^2+^ imaging was below the threshold for Ca^2+^ uncaging. Reproducible increases in [Ca^2+^]_i_ were detected over multiple uncaging events, and no increases in [Ca^2+^]_i_ were detected in non-loaded slices.

### Two-Photon Imaging of Astrocytic Endfoot for ROS Study

ROS production was monitored at 35°C by using the 5-(and-6)-chloromethyl l-2′,7′-dichlorodihydrofluorescein diacetate (CM-H_2_DCFDA), a dye sensitive to peroxynitrite 1 (ONOO-), hydrogen peroxide (H_2_O_2_), and hydroxyl radical (HO_; Molecular Probes). CMH_2_DCFDA is a non-fluorescent dye until the acetate groups are removed by intracellular esterases and oxidation occurs within the cell. The dye (50 μg) was freshly dissolved each day in 5 μL dimethyl sulfoxide (DMSO). The slices were loaded with 30 μM CM-H_2_DCFDA in the dark (45 min; 25°C; 0.005% [w/v] Pluronic F127). After loading, the slices were washed three times and then kept in aCSF for at least 30 min to ensure deacetylation. Probenecid (1.5 mM) was added to every solution used after loading to inhibit organic anion transporters that remove fluorescent dyes from the cytoplasm. ROS was imaged at 730 nm using the two photon microscope at the same condition as for Ca^2+^ imaging experiments. We assigned the threshold for ROS increases as 1.2 fractional fluorescence (F1/F0).

### Endfoot Ca^2+^ and ROS Analysis

F1/F0 values reflect the Ca^2+^ or ROS fluorescence intensity for a ROI in each image (F1) divided by a mean fluorescence value (F0) taken from an average of 15–20 images before stimulation. For some experiments, astrocytic endfoot Ca^2+^ concentrations were determined using the maximal fluorescence method as described earlier (Girouard et al., 2010). To summarize, ionomycin (407950, 10 μM; EMD Calbiochem) and 20 mM Ca^2+^ were immediately added to aCSF at the end of each experiment to obtain the maximal fluorescence (F_MAX_). The F_MAX_ value was measured within a ROI (15 × 15 pixels, or 1.8 × 1.8 μm) in the selected endfoot. Using this value and experimental parameters, the estimated [Ca^2+^]_i_ was calculated using Maravall’s formula (Maravall et al., 2000; Girouard et al., 2010).

### Electrophysiology Analysis

After 1 h recovery, the slices were transferred to a recording chamber and perfused continually with the oxygenated aCSF at the temperature of 32 ± 1°C. A glass microelectrode with the resistance of 2–3 MΩ filled with oxygenated aCSF inserted into radiatum layer of CA1 region. Excitatory postsynaptic potential (EPSP) was generated by stimulating the Schaffer collateral pathway using a stimulator (SEN-3301, Nihon Kohden, Japan). Stimulus pulses (0.1 ms duration) were delivered every 15 s. Signals were obtained using an Axoclamp 2B amplifier (Axon Instruments, Foster City, CA, United States), sampled at 20 kHz, and filtered at 10 kHz, and the output was digitized with a Digidata 1200 converter (Axon Instruments). Stability of baseline recordings was established by delivering single pulses (4/min, 0.1 ms pulse width) for 15 min prior to collection of data. (1) Basal synaptic transmission was assayed by determining input–output relations from extracellular field potential recordings in the stratum radiatum of CA1; the input was the peak amplitude of the fiber volley, and the output was the initial slope of the EPSP. EPSP slopes were evoked by testing stimulation of various intensities (0.1–1.1 mA). (2) Paired-pulse facilitation (PPF) was induced by double stimuli with interpulse interval (IPI) of 25–200 ms. The value of paired-pulse ratio (PPR) is expressed by the second EPSP slope relative to the first EPSP slope. (3) Long-term potentiation (LTP) was induced by four tetani delivered 10 s apart, each at 100 Hz for 1 s after a 20 min baseline period. After delivering tetani, the EPSP slopes were recorded for a further period of 60 min. LTP was determined if the EPSP slopes were larger by 20% compared to the baseline.

### Statistical Analysis

All results are presented as mean ± SEM. Two-way (with repeated measures when appropriate) ANOVA and Bonferroni’s multiple comparison test were performed to compare differences among groups. The two-tailed unpaired Student’s *t*-test was performed for comparison between two-groups. Differences at *p* < 0.05 were considered statistically significant.

## Results

### Hippocampal Neurovascular Coupling Is Impaired in APP Mice

We first determined hippocampal CBF increases in response to *in vivo* electrical stimulation of Schaffer collaterals in APP and WT mice. Stimulus-induced hippocampal CBF increase in the CA1 area was significantly reduced in APP mice compared with age-matched WT mice (Figure 1B, *p* < 0.0001), consistent with our previous report of impaired NVC in the cerebral cortex of similarly aged APP mice (Tong et al., 2009).

Vascular loss is a hallmark of the microvascular pathology in AD (Bailey et al., 2004) and may explain, at least in part, the attenuated CBF increase. Staining of vessel basement membrane with collagen IV or endothelial cells with Ly6c showed that both vascular surface area and vessel diameter were comparable between WT and APP mice of the same age (Figure 1C), indicating no apparent vascular density loss or diameter changes in APP mice at that age.

Then, we investigated the mechanisms underlying impaired NVC using acute hippocampal slices. The mean diameter of arterioles at rest was 10.5 μm with a range of 7.5–15 μm. To guarantee that the impaired vascular responses were truly related to EFS in our experimental conditions (33% of experiments using EFS induced neither vascular nor astrocytic endfoot response; data not shown), we defined over 20% increase in Ca^2+^ fluorescence post stimulation as a successful EFS. This threshold was determined from our previous experiments which showed that the coefficient of variation of resting Ca^2+^ is below 20% (Boily et al., 2021). Imaging in the absence of EFS did not induce any changes in diameter or endfoot Ca^2+^ (Boily et al., 2021).

**FIGURE 2:**
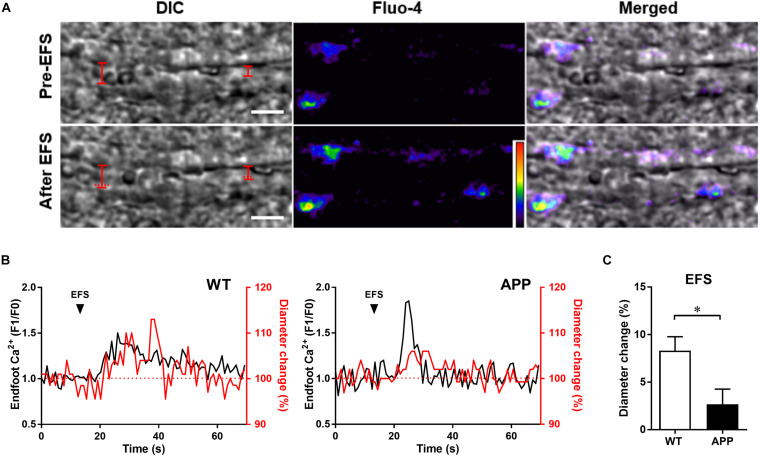
Vascular and astrocytic Ca^2+^ responses to electrical field stimulation (EFS) are impaired in hippocampal slices of APP mice. **(A)** Example of simultaneous recording of changes in arteriolar diameter and astrocytic endfoot Ca^2+^ increases in response to EFS in an hippocampal slice from a WT mouse. Left panel: representing infrared differential interference contrast (IR-DIC) images of an hippocampal arteriole obtained Pre- and after EFS (scale bar, 10 μm). The vascular diameter is labeled by red solid lines or dashed lines (indicate the pre-EFS position of the vessel wall). Middle panel: pseudocolor Fluo-4 AM fluorescence representing Ca^2+^ in astrocytic endfeet surrounding an arteriole in an hippocampal slice. Right panel: Merged pictures from left and middle panels. **(B)** Representative traces of EFS induced changes in endfoot Ca^2+^ (black) and arteriolar diameter (red) in WT and APP mice. **(C)** Hippocampal vascular dilation evoked by EFS in APP mice is reduced compared to WT controls (^∗^*p* < 0.05; two-tailed unpaired *t*-test; *n* = 12–13). Analysis of Ca^2+^ responses to EFS are presented in Figure 3. F1/F0: fractional fluorescence.

In addition, 68% (data not shown) of astrocytic endfoot Ca^2+^ increases were accompanied by vascular responses in hippocampal slices of WT mice. EFS that triggered astrocytic endfoot Ca^2+^ increases also induced vascular dilation in both APP and WT mice (Figures 2A,B). In accordance with *in vivo* results, the amplitude of vascular responses to EFS in acute hippocampal slices was significantly reduced in APP mice compared with WT mice (Figure 2C, *p* < 0.05).

### The Astrocytic Endfoot Ca^2+^ Increase in Response to Neuronal Activity Is Attenuated in APP Mice

**FIGURE 3:**
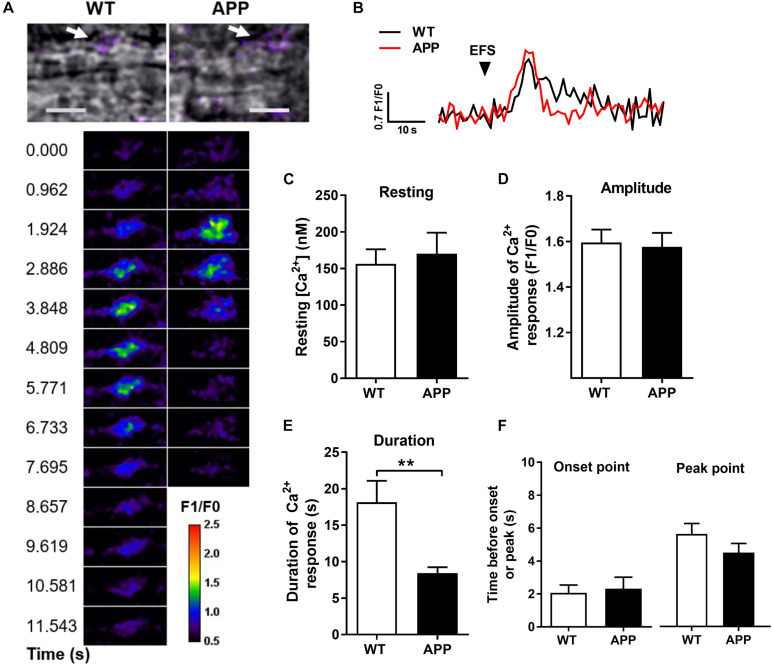
Astrocytic Ca^2+^ response to electrical field stimulation (EFS) in the hippocampal slices is shorter in APP mice. **(A)** Infrared differential interference contrast (IR-DIC) images of an hippocampal arteriole in brain slices from WT and APP mice. Arrows show the analyzed astrocytic endfeet (upper panel, scale bars, 10 μm). Pseudocolor Fluo-4 AM fluorescence representing time-lapse images of Ca^2+^ elevation in astrocytic endfeet surrounding arterioles in brain slices from WT and APP mice following EFS (lower panel, pseudocolors legend unit corresponds to F1/F0). **(B)** Representative time-lapse traces of Ca^2+^ elevation within an endfoot in response to EFS. **(C)** Resting astrocytic endfeet Ca^2+^ concentrations in hippocampal slices and **(D)** amplitude of astrocytic endfeet Ca^2+^ responses to EFS are comparable in WT and APP mice. In contrast, the duration of Ca^2+^ elevation **(E)** is shorter in APP mice compared to WT controls, but not the time before the onset or the peak of Ca^2+^ elevation **(F)** in response to EFS (**p < 0.01; two-tailed unpaired *t*-test; *n* = 14–15).

The resting level of endfoot Ca^2+^ and the amplitude of astrocytic Ca^2+^ responses to EFS were similar in both APP and WT mice (Figures 3A–D). Neuronal activity-dependent [Ca^2+^]_i_ elevations in astrocytic endfeet occurred within 3 s after the onset of the stimulation and reached a peak after an average of 5.6 ± 0.64 (*n* = 15) and 4.5 ± 0.56 (*n* = 13) s in WT and APP mice, respectively. There was no difference for the period before the onset or peak point of astrocytic Ca^2+^ increases between WT and APP mice. However, the duration of astrocytic endfoot Ca^2+^ elevation was shorter in APP mice compared with WT (Figures 3E,F, *p* < 0.01).

### Gliovascular Ca^2+^ Signaling Dependent Pathway Is Impaired in APP Mice

**FIGURE 4:**
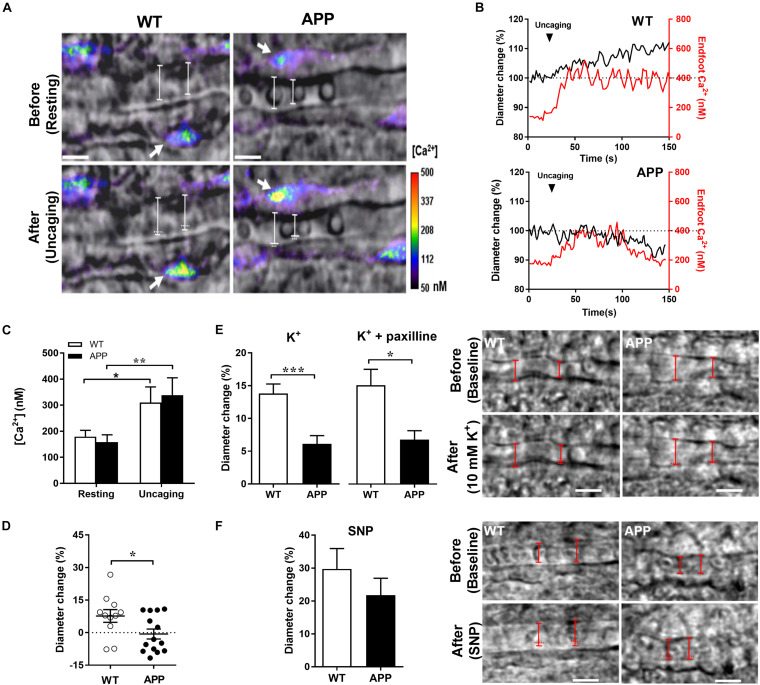
Impaired gliovascular coupling in the hippocampal slices of APP mice. **(A)** Infrared differential interference contrast (IR-DIC) images before (resting) and after two photon photolysis of caged Ca^2+^ within an astrocyte endfoot surrounding arterioles in hippocampal slices. Ca^2+^ uncaging was achieved in slices loaded with DMNP-EDTA (10 μM, 60 min) by scanning a region of interest within an endfoot at a higher (6×) laser intensity for ∼0.5 s. The resulting elevations in endfoot Ca^2+^ (range, 200–500 nM) caused arteriole dilation in slices from WT controls but constriction in slices from APP mice. Arrows indicate target endfeet of Ca^2+^ uncaging. The vascular diameter is labeled by white solid lines whereas white horizontal dots indicate the previous position of the vessel wall before Ca^2+^ uncaging (Scale bars, 10 μm). **(B)** Time course traces of changes in endfoot Ca^2+^ (red) and arteriole diameter (black) in response to Ca^2+^ uncaging in WT and APP mice. **(C)** Astrocytic endfoot Ca^2+^ before (resting) and at its peak after Ca^2+^ uncaging and **(D)** the percentage of changes in arteriolar diameter evoked by Ca^2+^ uncaging in the same group of hippocampal slices from WT and APP mice. **(E)** Vascular response to 10 mM K^+^ perfusion in hippocampal slices from APP mice pre-incubated or not with paxilline (1 μM) (left panel). Infrared differential interference contrast (IR-DIC) images before (baseline) and after a 10 mM K^+^ perfusion of an hippocampal arteriole from WT and APP mice. The vascular diameter is labeled by red solid lines whereas the horizontal red dots indicate the pre-K^+^ incubation position of the vessel wall (right panel, scale bars, 10 μm). **(F)** Sodium nitroprusside (SNP) induced a small, non-significant decrease of arteriole dilation in slices of APP mice (left panel). Infrared differential interference contrast (IR-DIC) images before (baseline) and after SNP perfusion of an hippocampal arteriole from WT and APP mice. The vascular diameter is labeled by red solid lines whereas the horizontal red dots indicate the Pre-SNP incubation position of the vessel wall (right panel, scale bars, 10 μm). **(C)** *p < 0.05, **p < 0.01; two-way ANOVA repeated measures followed by Bonferroni correction, *n* = 6–8; **(D,E)** *p < 0.05, ***p < 0.001; two-tailed unpaired *t*-test, *n* = 11–14).

We next asked whether the vascular responses to astrocytic Ca^2+^ increases are impaired in cerebral blood vessels with no apparent vascular amyloidosis from 6-month-old APP mice (Tong et al., 2009). We thus increased astrocytic Ca^2+^ levels similarly in APP and WT mice using two photon photolysis of caged Ca^2+^ DMNP-EDTA. Uncaging generated modest increase of endfoot Ca^2+^ in a range of 200–600 nM, which are concentrations known to induce arteriolar dilation (Girouard et al., 2010). The Ca^2+^ increase induced by Ca^2+^ uncaging in endfoot was similar in APP and WT mice, showing long lasting elevated Ca^2+^, but different corresponding vascular responses (Figures 4A–C). In the hippocampal slices of APP mice, the arterioles shifted more toward constriction compared with WT mice (Figure 4D, *p* < 0.05). This inability to dilate may be explained by a higher level of extracellular perivascular K^+^ or attenuated arteriolar dilation response to 10 mM K^+^ or to an NO donor in APP mice. Indeed, the vascular responses to K^+^ in the presence or absence of the large conductance of Ca^2+^ activated K^+^ (BK) channel blocker paxilline were attenuated in APP mice compared to their WT counterpart (Figure 4E, *p* < 0.05). In contrast, dilations to the NO donor, SNP, or constrictions to the thromboxane A2 receptor agonist, U46619 were not significantly different in APP mice compared to WT controls (Figure 4F and Supplementary Figure 1). These data suggest that gliovascular coupling downstream of astrocytic enfoot Ca^2+^ increase and BK channels is impaired in adult APP mice.

### Increased Amplitude of Spontaneous Ca^2+^ Oscillations in Astrocytic Endfeet of APP Mice

**FIGURE 5:**
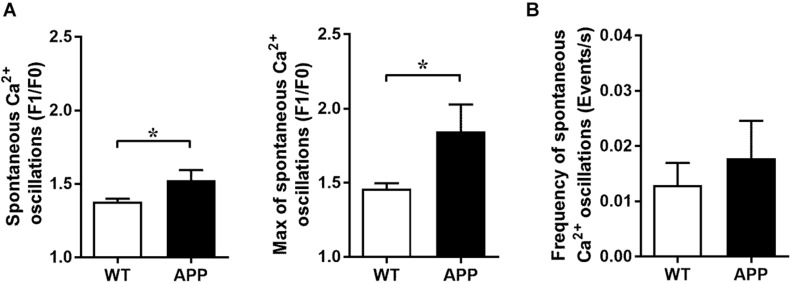
Increased amplitude of spontaneous Ca^2+^ oscillations in astrocytic endfeet of APP mice. **(A)** Averaged (left panel) and maximal amplitude (right panel) and **(B)** frequency of spontaneous Ca^2+^ oscillations in astrocytic endfeet of hippocampal slices from WT and APP mice (*p < 0.05; two-tailed unpaired *t*-test; *n* = 4–13). F1/F0: fractional fluorescence.

The frequency and amplitude of spontaneous astrocytic Ca^2+^ oscillation events may impact Ca^2+^ increases to a stimulus. These events were defined as increases of fluorescence of more than 30% (determined from the coefficient of variability of the baseline intensity) from the baseline intensity (ΔF/F). As expected, the maximal and averaged amplitudes of spontaneous Ca^2+^ oscillations in astrocytic endfeet, not their frequency, were significantly increased in APP mice compared with WT mice (Figure 5, *p* < 0.05). Since the elevated spontaneous Ca^2+^ oscillation may be due to high levels of Aβ peptide or Aβ plaques, we verified whether blood vessel and astrocytic endfeet are located in proximity of Aβ plaques (Supplementary Figure 2).

### Impaired Vascular Responses Are Highly Associated With ROS Concentrations

**FIGURE 6:**
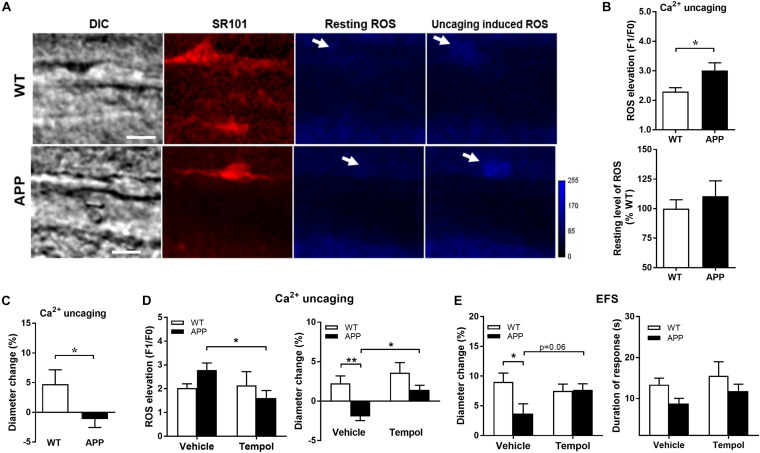
Ca^2+^ uncaging induces a higher level of ROS production in astrocytic endfeet in APP mice. **(A)** Representative images of infrared differential interference contrast (IR-DIC), SR101 (astrocytes), resting ROS and Ca^2+^uncaging induced ROS in brain slices from WT and APP mice. Arrows indicate targeted endfeet of Ca^2+^ uncaging (Scale bars, 10 μm). **(B)** Astrocytic endfeet ROS production and **(C)** vascular diameter after Ca^2+^ uncaging in hippocampal slices from APP mice compared with WT (*p < 0.05; two-tailed unpaired *t*-test; *n* = 12–16). **(D)** Tempol, through reducing the Ca^2+^ uncaging-induced astrocytic ROS production (left panel), prevented the impaired vascular response to Ca^2+^ uncaging (right panel) in APP mice (*p < 0.05; **p < 0.01; two-way ANOVA followed by Bonferroni correction, *n* = 9–15). **(E)** Tempol also prevented the reduced vascular responses to EFS (left panel) without modifying the duration of the response (right panel) (*p < 0.05; two-way ANOVA followed by Bonferroni correction, *n* = 9–15).

We then verified whether endfoot Ca^2+^ uncaging increases astrocytic endfoot ROS levels and, if so, whether it correlates with the amplitude of vascular responses. As shown in Figure 6A, astrocytic endfoot ROS levels were increased following Ca^2+^ uncaging but more importantly in APP mice compared with WT littermates while the resting levels of ROS in astrocytic endfeet were similar between WT and APP mice (Figure 6B, *p* < 0.05). The dilatatory response in APP mice was significantly reduced (Figure 6C, *p* < 0.05).

We further pre-incubated brain slices with Tempol, a superoxide dismutase mimetic, to prevent ROS production induced by Ca^2+^ uncaging. Notably, ROS elevation observed in APP mice was significantly reduced to levels comparable to those of WT mice in the presence of Tempol, while Tempol did not further decrease ROS in WT mice (Figure 6D). Tempol reversed the polarity of the vascular responses to Ca^2+^ uncaging in APP mice from a tendancy to constriction to a small dilation (Figure 6D, *p* < 0.05, *p* < 0.01). In order to exclude a potential effect of laser Ca^2+^ uncaging damage to astrocytic enfeet and to induce a more physiological stimulation of vascular response, we tested the effect of Tempol on EFS induced vascular responses. Tempol normalized the vascular response to EFS and the duration of astrocytic endfoot Ca^2+^ elevations in APP mice (Figure 6E, *p* < 0.05). The above results confirmed that astrocytic ROS contribute to NVC impairment in APP mice.

### Impaired Hippocampal Synaptic and Vascular Function in APP Mice

**FIGURE 7:**
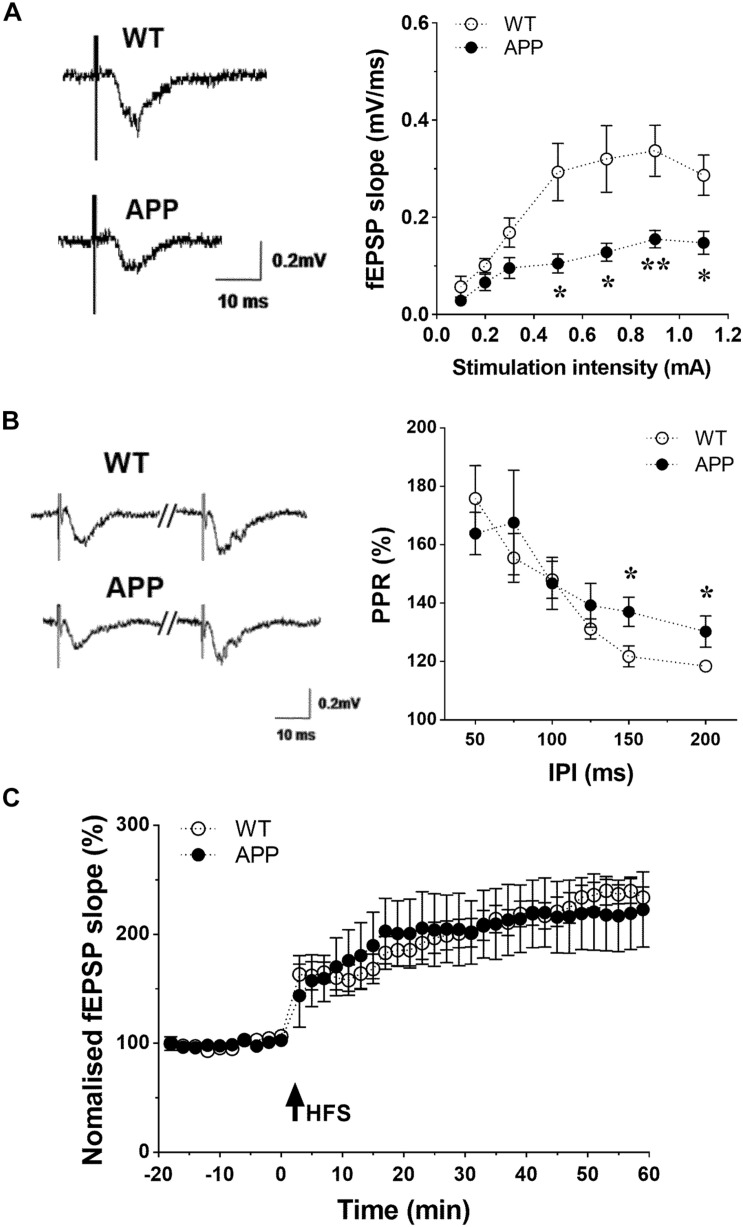
Impaired hippocampal synaptic function in APP mice. **(A)** Basic synaptic transmission impaired in hippocampal CA1 region of APP mice. Input/output curve: Slopes of fractional changes in excitatory postsynaptic potential (EPSPs) were plotted against stimuli ranging from 0.1 to 1.1 mA. **(B)** Paired-pulse ratios (PPR, %) of fEPSPs were plotted against various interpulse intervals (IPI) ranging from 50 to 200 ms. PPR at the Schaffer collaterals-CA1 synapse were unchanged with IPI from 50 to 125 ms, but enhanced with IPI from 150 to 200 ms in APP mice compared to WT. **(C)** Induction of LTP by high frequency stimulations (HFS)-100p (100 Hz at 100 pulses) in hippocampal slices obtained from WT and APP mice (*p < 0.05, **p < 0.01; two-tailed unpaired *t*-test *n* = 6–8).

To determine whether synaptic transmission was also disturbed at the level of the Schaffer collaterals-CA1 pathway, fractional changes in excitatory postsynaptic potential (EPSP) were plotted against stimulation intensities. The input-output curve revealed that the EPSP in APP mice was significantly lower between 0.5 and 1.2 mA (Figure 7A, *p* < 0.05, *p* < 0.01). The paired-pulse ratio (PPR), a form of calcium-dependent short term plasticity, with interpulse interval (IPI) ranging from 50 to 200 ms was measured to analyze the potential for presynaptic glutamate release. The value of IPI between 50 and 125 ms did not differ between APP and WT mice (Figure 7B, *p* < 0.05). However, at IPI from 150 to 200 ms, the PPR was significant higher in APP mice compared to their WT littermates, indicating a prolonged increased pre-synaptic glutamate release. The induction of Schaffer collaterals-CA1 LTP was examined by delivering a conditioning stimulation. As shown in Figure 7C, the high frequency conditioning stimulus (4 times 100 pulses at 100 Hz) induced an increase of EPSP of approximately 110% lasting over 60 min in hippocampal slices from both APP and WT mice. Interestingly, we found that APP mice could be divided into two subgroups with 4–6 mice showing an impaired LTP in 60 min after HFS while the rest showed improved LTP (results not shown).

## Discussion

This study shows, for the first time, specific impairments of hippocampal neurogliovascular communications in APP mice. We demonstrated that, in APP mice compared to WT controls, (*i*) the *in vivo* CBF increase in response to Schaffer collaterals stimulation is reduced, and (*ii*) the *ex vivo* vasodilation in response to neuronal stimulation or astrocytic Ca^2+^ elevation is also diminished. These observations may be explained, at least in part, by an attenuated fEPSP slope and PPR, shorter astrocytic endfoot Ca^2+^ increases and a decreased vascular reactivity to K^+^. Scavenging ROS with Tempol in APP mice normalized the vascular responses to neuronal stimulation and astrocytic Ca^2+^ elevations.

While many studies have investigated NVC in the somatosensory cortex (Park et al., 2004, 2020; Ongali et al., 2010; Duncombe et al., 2017; Royea et al., 2020) of APP mice, the present study establishes, both *in vivo* and *ex vivo*, that NVC is also impaired in the hippocampus of APP mice. Considering that, in human, Aβ deposition occurs mainly in the enthorinal cortex and hippocampus (Ulrich, 1985; Vogel et al., 2021), it is crucial to understand the mechanisms leading to neurodegeneration in these regions. In the present study, APP J20 mice expressed both the human APP familial Swedish and Indiana mutations and are characterized by the presence of Aβ plaques in the hippocampus at 6 months of age (Aucoin et al., 2005). We also confirmed the presence of Aβ plaques in the vicinity of astrocytes and blood vessels of the hippocampus.

NVC is, at least partially, dependent on astrocytic Ca^2+^ rises following neuronal activation (Zonta et al., 2003a; Filosa et al., 2004; Mulligan and MacVicar, 2004; Mishra et al., 2016; Gu et al., 2018). In the present study, we observed an increased amplitude of spontaneous Ca^2+^ oscillations in astrocytic endfeet with a shorter duration of astrocytic Ca^2+^ elevations in response to EFS. Increased in spontaneous astrocytic Ca^2+^ activity has also been reported in APP/PS1 mice (Kuchibhotla et al., 2009).

This increase in spontaneous activity seems to be independent of changes in neuronal activity (Kuchibhotla et al., 2009). Actually, acute or chronic exposure of isolated astrocytes to Aβ has been shown to enhance spontaneous intracellular Ca^2+^ transient signaling (Haughey and Mattson, 2003; Lim et al., 2013; Alberdi et al., 2013; Lee et al., 2014). This increase in Ca^2+^ partially originates from intracellular sources such as the endoplasmic reticulum (Toivari et al., 2011). In addition, Aβ interacts with several types of receptors in astrocytes leading to Ca^2+^ entry. These receptors include the purinergic receptor P2Y1 (Delekate et al., 2014), nicotinic receptors (α7-nAchRs) (Xiu et al., 2005; Lee et al., 2014) and glutamate metabotropic receptors (mGluR) (Grolla et al., 2013; Ronco et al., 2014).

The increased amplitude of spontaneous astrocytic Ca^2+^ may be responsible for the decreased duration of astrocytic Ca^2+^ elevations in response to neuronal activation. Indeed, since cytoplasmic Ca^2+^ regulation of inositol 1,4,5-trisphosphate receptors (IP_3_Rs) is biphasic (Bezprozvanny et al., 1991; Foskett et al., 2007), relatively high resting [Ca^2+^] may dampen the amplitude and/or the duration of the IP_3_Rs-dependent astroglial [Ca^2+^] increases. The decreased duration of astrocytic Ca^2+^ elevations in response to neuronal activation may also result from a decreased rate of exocytosis and fraction of released vesicles, as was observed in APP mice. Indeed, these parameters are known to correlate with Ca^2+^ mobilization in cultured astroglia (Kreft et al., 2004; Pryazhnikov and Khiroug, 2008). In turn, decreased duration of astrocytic Ca^2+^ elevations may have an impact on synaptic function (gliotransmitter release, synaptic plasticity, and integrity) (Min and Nevian, 2012).

Resting astrocytic Ca^2+^ controls tonic vasodilation independently of neuronal activity (Rosenegger et al., 2015). However, in the present study, the vascular tone does not seem to differ between WT and APP mice since the level of preconstriction induced by a thromboxane receptor agonist was the same in these groups (Blanco et al., 2008). The link between spatiotemporal characteristics of astrocytic Ca^2+^ increases and the amplitude and duration of the vascular response remains to be further investigated (Sharma et al., 2020).

To better understand how the gliovascular communication is impacted in APP mice, Ca^2+^ was uncaged in the astrocytic endfeet to induce comparable Ca^2+^ increases. Interestingly, the vascular responses to Ca^2+^ uncaging was shifted toward constriction or smaller dilations in APP mice compared with WT controls suggesting a change in vascular reactivity to astrocytic-derived vasomediators. Astrocytic Ca^2+^ increases induce the release of various vasoactive molecules (Girouard and Iadecola, 2006). These include arachidonic acid metabolites such as prostaglandin E2 (PGE2) (Zonta et al., 2003b; Takano et al., 2006; Gordon et al., 2008; Mishra et al., 2011; Hall et al., 2014) and epoxyeicosatrienoic acids (EETs) (Alkayed et al., 1997; Metea and Newman, 2006b), ions such as K^+^ (Filosa et al., 2004), and metabolic by-products such as lactate (Gordon et al., 2008) and adenosine (Xu and Pelligrino, 2007; Vetri et al., 2011). Astrocytes could also release arachidonic acid which is metabolized into the vasoconstrictor hydroxyeicosatetraenoic acid (20-HETE) in vascular smooth muscle cells (Mulligan and MacVicar, 2004; Metea and Newman, 2006a; Mishra et al., 2011; Hall et al., 2014). Since K^+^ exerts a biphasic effect on vascular responses, low concentrations leading to dilation and higher concentrations causing constriction, we hypothesized that astrocytes in APP mice may release more K^+^. However, the selective BK-channel blocker, paxilline did not modify the vascular response to K^+^, while the vascular responses to K^+^ alone are attenuated. This effect seems to be specific to K^+^ since vascular responses to the NO donor, SNP, or the thromboxane A_2_ receptor agonist, U46619 did not differ between WT and APP mice. These results suggest that the inward rectifier K^+^ (K_ir_) channels may be altered in APP mice. Indeed, it was demonstrated that impairment in NVC in a mouse model of familial AD (5xFAD) is attributable to reduced activity of capillary K_ir_2.1 channels (Mughal et al., 2021). K_ir_ channel function was also altered in cerebrovascular endothelial cells of the triple transgenic AD model (mutations in the PS1, APP, and tau genes) (Hakim and Behringer, 2020). Capillaries K_ir_2.1 channels are involved in NVC by translating local elevation of K^+^ into upstream arteriolar dilations (Longden et al., 2017). Overall, these data suggest that the K_ir_ channel-dependent vascular response to astrocyte-derived K^+^ is impaired in APP mice. However, this does not exclude the possibility of a change in the concentration or nature of vasomediators derived from astrocytes.

Using the superoxide anion mimetic, Tempol, we demonstrated that ROS are involved in the impaired vascular responses to neuronal and astrocytic activation but not in the decreased duration of astrocytic Ca^2+^ responses to neuronal activity. The impact of ROS on NVC had been demonstrated in many models. In APP mice, it was demonstrated that cerebrovascular oxidative stress originates from the action of Aβ peptide on the innate immunity receptor CD36 on perivascular macrophages, which in turn, activates a Nox2-containing NADPH oxidase. This Aβ-induced vascular oxidative stress resulted in impaired NVC (Tong et al., 2009; Park et al., 2017) and endothelial-dependent dilatory function (Zhang et al., 2013). Nevertheless, although it had been suggested that ROS may block or knockdown K_ir_2.1 channels (Zhang et al., 2019), the cause-effect relationship between ROS and impaired K_ir_ vascular dependent responses in APP mice remains to be established.

In conclusion, we have demonstrated that hippocampal NVC is altered at many levels of the neurovascular unit in APP mice and that an acute antioxidant treatment normalizes NVC as well as the vascular response to astrocytic Ca^2+^ pathway activation. These data support the inclusion of antioxidants in therapies aiming to preserve the neurovascular unit in AD.

## Data Availability Statement

The original contributions presented in the study are included in the article/Supplementary Material, further inquiries can be directed to the corresponding author/s.

## Ethics Statement

The animal study was reviewed and approved by the Canadian Council on Animal Care, Committee on Ethics of Animal Experiments and Animal Research: Reporting of *in vivo* Experiments of the Université de Montréal and the Montreal Neurological Institute at McGill University.

## Author Contributions

LL, X-KT, EH, and HG contributed to conception and design of the study. LL, X-KT, and DV performed the research. LL, X-KT, DV, and HG analyzed the data. LL, X-KT, MH, DV, and HG wrote the manuscript. All authors contributed to the article and approved the submitted version.

## Conflict of Interest

The authors declare that the research was conducted in the absence of any commercial or financial relationships that could be construed as a potential conflict of interest.

## Publisher’s Note

All claims expressed in this article are solely those of the authors and do not necessarily represent those of their affiliated organizations, or those of the publisher, the editors and the reviewers. Any product that may be evaluated in this article, or claim that may be made by its manufacturer, is not guaranteed or endorsed by the publisher.
