# Supplementary material for: Impaired Hippocampal Neurovascular Coupling in a Mouse Model of Alzheimer’s Disease
Source: Front Physiol. 2021 Aug 12;12:715446. doi: 10.3389/fphys.2021.715446 (PMC8406685; doi:10.3389/fphys.2021.715446)
Supplement: Supplementary file 1 [file Data_Sheet_1.PDF]

Supplemental files

Impaired hippocampal neurovascular coupling in a mouse model of Alzheimer's disease

[1,2]Lin Li PhD, [3]Xin-Kang Tong PhD, [1,2,4]Mohammadamin Hosseini Kahnouei MD, [1,4]Diane Vallerand Bacc, [3]Edith Hamel PhD and [1,2,4,5]Hélène Girouard PhD

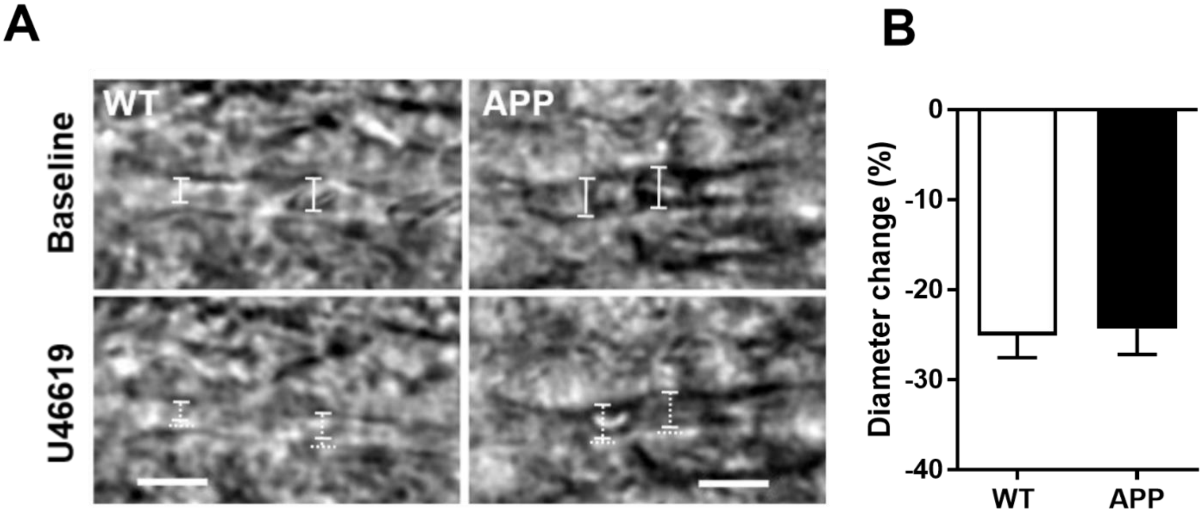

Supplemental Figure 1. Vascular diameter changes in response to the thromboxane A2 receptor agonist, U46619, are similar in hippocampal slices from WT and APP mice. (A) Representative images of infrared differential interference contrast (IR-DIC). The vascular diameter is labelled by white solid lines or dashed lines (indicate the U46619 incubation of the vessel wall). (B) Bar graph of vascular diameter in response to U46619. The brain slices were incubated with U46619 (150nM; 20 min) to preconstrict arterioles at a physiological level (scale bars=10 μm, n=20-21).

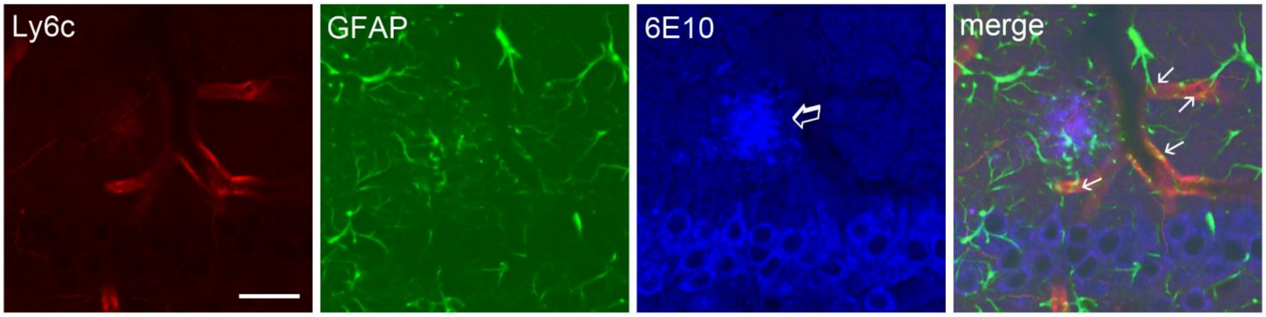

Supplemental Figure 2: Relationships between blood vessels, activated astrocytes, and Aβ plaques in the hippocampal slices of APP mice. Triple immunostaining on hippocampal blood vessels (Ly6c, red) showing the presence of astroglial endfeet (GFAP, green) in proximity of an Aβ plaque (6E10, blue, open arrow). A merge image (right panel) shows astroglial endfeet contacting the vessel wall (arrows). Bar = 20µm, n=20-21.
